# Current News and Opinion

**Published:** 1882-06

**Authors:** 


### CURRENT NEWS AND OPINION.

The Indiana State Dental Association will meet in Indianapolis on the last Tuesday in June.

The Connecticut Valley Dental Society will hold its summer meeting at Amherst, Mass., June 29 and 30.

THE American Dental Convention will hold its annual meeting at Saratoga Springs on the 8th of August.

THE SOUTHERN Dental Association will hold its next annual meeting in Baltimore, Md., August 8th, 1882.

DR. FRANK H. COBURN, a promising young dentist of Nashua, N. H., died April 15, in the 23d year of his age.

THE PENNSYLVANIA State Dental Society meets July 25, at Williamsport, Pa., and continues in session three days.

CHIAN turpentine, as a cure for cancer, is taking its departure—like its predecessor, cundurango, "down the back entry of time."

DR. JOHN P. GRAY, who was recently shot and dangerously wounded by a lunatic with a "Divine Commission," is steadily improving.

THE AMERICAN Dental Association will meet in Cincinnati on the first Tuesday in August. The Highland House has been secured for the sessions.

AN OINTMENT made with animal charcoal (and vaseline) is claimed to be a specific in acute eczema, by Onocool Chunder Chatterjee in the *Indian Medical Gazette.*

A PHOTOGRAPHIC group of a large number of the members in attendance at the late International Medical Congress has been published by Mr. Barraud of London.

ACCORDING to Rabuteau, who determined the fact by auto-experimentation, sulpho-phenate and sulpho-cresylate of soda are mildly purgative in doses of 25 to 30 grams (about 6–8 drams).

THE homœopathists of Massachusetts are making an effort to have a State asylum for the insane placed in their hands. If the experiment, when undertaken, succeeds—*similia similibus curantur.*

DR. MURRELL has tested nitro-glycerine in three severe cases of angina pectoris with a success quite equal to that afforded by nitrite of amyl. Dose, one minim of a one per cent solution every three hours.

DR. ALFRED WILTSHIRE has reported a case of total inversion of the vagina and prolapsus of the uterus from sudden effort. The patient was a housemaid, 26 years of age, and had given birth to a child nine months before.

Dr. C. W. BÖDECKER begins a series of interesting papers on the minute anatomy, physiology, pathology and therapeutics of the dental pulp in the *Dental Cosmos* for June. We will give an abstract of the paper when completed.

THE MICHIGAN State Medical Society met at Ypsilanti on May 10 and

11. Dr. G. W. Topping, of De Witt, was elected President for the current year, and Dr. G. E. Ranney, of Lansing, the present efficient secretary, was re-elected.

Dr. J. S. Billings said very wisely in his address to the graduating class of Bellevue Hospital Medical College : "In matters of ethics your first duty would be for each to look after his own ethics, before he gives himself much trouble about the ethics of his neighbor."

The Medical Society of the State of West Virginia held its 15th annual meeting in Wheeling on May 24 and 25. From the address of the President, Dr. James E. Reeves, we learn that the total number of legally qualified medical practitioners in the State is 901, only 442 of whom are graduates of reputable medical colleges.

Joffroy and Pitres have reported cases of rapid exfoliation of the toe-nails in advanced locomotor ataxy. The cases are abstracted in the *Neurologisches Centralblatt*, No. 8, 1882. The falling of the nails was not in consequence of injury, and was not accompanied by suppuration. The nails were rapidly reproduced.

The new Antiseptic, boro-glyceride, of which we give an account in our *Popular Science Department*, has been successfully used by Mr. Richard Barwell, as an antiseptic in surgery. He places a few folds of lint soaked in the solution upon the wound. It is unirritating, and healing takes place in a perfect manner. The dressing is exceedingly simple.

Alcohol, instead of alcoholic liquors (whiskey, brandy, wine, etc.) is recommended by Dr. Lewis D. Mason where a stimulant is indicated. The dose can be exactly adjusted, the taste readily disguised, adulterations avoided, and little danger exists of cultivating a taste for intoxicating drinks. It is also cheaper. The suggestion is, we believe, originally due to Dr. B. W. Richardson and deserves a fair trial.

Complaints have of late years been often made in Germany of the fast increase of shortsightedness among young people at school, and similar ones are now heard in France. A committee appointed to investigate the subject have reported that the cause of the evil lies in the school-books, which are printed in fine type on white paper. They suggest that larger-faced type be employed hereafter, and that white letters be printed upon tinted paper.

Pilocarpine is employed in the Maternity Hospital of Brussels in puerperal eclampsia (*Journ. de Bruxelles*, Oct. 1881). Its effects are most beneficial in cases of generalized œdema. When there exists profound coma during the whole interval of the attacks, much more prompt return of the intellectual faculties is obtained than by any other means used for this purpose. The dose is $\frac{1}{4}$ to $\frac{1}{2}$ grain hypodermically. The writer of this note used fluid extract of Jaborandi, $\frac{1}{2}$ dram every fifteen minutes until free perspiration followed, with the happiest effect in such cases, over a year ago.

IN THE *Gaceta Med. Prov. Venete,* M. Cavazzini claims to have performed resection of the stomach in 1874, seven years before Billroth's first operation on the human subject. The patient was a woman, 27 years of age, who had suffered for over a year from a growth apparently situated in the abdominal walls directly over the stomach. The symptoms becoming urgent it was decided to remove the growth with the knife. It was found that the stomach was involved in the growth, and a portion of its walls had to be removed in order to extirpate the tumor. The patient recovered and died five years afterward of consumption.

